# An integrated approach to characterize transcription factor and microRNA regulatory networks involved in Schwann cell response to peripheral nerve injury

**DOI:** 10.1186/1471-2164-14-84

**Published:** 2013-02-06

**Authors:** Li-Wei Chang, Andreu Viader, Nobish Varghese, Jacqueline E Payton, Jeffrey Milbrandt, Rakesh Nagarajan

**Affiliations:** 1 Department of Pathology and Immunology, Washington University School of Medicine, 660 South Euclid Ave, St. Louis, MO 63110, USA; 2 Department of Genetics, Washington University School of Medicine, 660 South Euclid Ave, St. Louis, MO 63110, USA

**Keywords:** Transcriptional regulatory network, MicroRNA regulatory network, Myelination, Schwann cells

## Abstract

**Background:**

The regenerative response of Schwann cells after peripheral nerve injury is a critical process directly related to the pathophysiology of a number of neurodegenerative diseases. This SC injury response is dependent on an intricate gene regulatory program coordinated by a number of transcription factors and microRNAs, but the interactions among them remain largely unknown. Uncovering the transcriptional and post-transcriptional regulatory networks governing the Schwann cell injury response is a key step towards a better understanding of Schwann cell biology and may help develop novel therapies for related diseases. Performing such comprehensive network analysis requires systematic bioinformatics methods to integrate multiple genomic datasets.

**Results:**

In this study we present a computational pipeline to infer transcription factor and microRNA regulatory networks. Our approach combined mRNA and microRNA expression profiling data, ChIP-Seq data of transcription factors, and computational transcription factor and microRNA target prediction. Using mRNA and microRNA expression data collected in a Schwann cell injury model, we constructed a regulatory network and studied regulatory pathways involved in Schwann cell response to injury. Furthermore, we analyzed network motifs and obtained insights on cooperative regulation of transcription factors and microRNAs in Schwann cell injury recovery.

**Conclusions:**

This work demonstrates a systematic method for gene regulatory network inference that may be used to gain new information on gene regulation by transcription factors and microRNAs.

## Background

Schwann cells (SCs), the main glia cells in the peripheral nervous system, are among a limited number of mammalian cells capable of dedifferentiation. The ability of SCs to dedifferentiate is critical to their role in supporting peripheral nerve regeneration. Following peripheral nerve injury, SCs mount a regenerative response involving coordinated dedifferentiation, proliferation and redifferentiation that supports axonal regrowth and helps restore peripheral nerve function [1]. Like other cellular processes tightly coupled with cell fate determination and developmental timing control, this SC injury response requires precise spatiotemporal regulation of gene expression. This is achieved via an intricate transcriptional program that maintains the balance between positive and negative regulators of SC differentiation [2]. In addition to transcriptional control, recent studies have shown that SC myelination [3-5] and response to injury [6] are also post-transcriptionally modulated by microRNAs (miRNAs).

Although the role of individual TFs that regulate SC myelination has been investigated, cooperation and interaction among different TFs involved in the response of SCs to nerve damage remain largely unknown. More importantly, how miRNAs integrate into the genetic program of TFs to modulate SC gene expression remains unclear. A comprehensive delineation of the TF and miRNA regulatory network underlying the SC injury response may shed light on fundamental aspects of SC biology. This information could also help fulfill the therapeutic potential of modulating the SC injury response in a number of neurodegenerative diseases characterized by peripheral axonopathy.

Systematically inferring TF and miRNA regulatory networks is difficult to achieve by experimental methods and has motivated development of computational approaches. Computational tools have been created to construct TF and miRNA regulatory networks using information such as gene expression profiling, miRNA expression profiling, and predicted TF and miRNA binding sites [7-11]. However, most of these tools utilized a subset of these data and few studies have combined all of these datasets to infer TFs and miRNA regulatory networks. For example, MIR@NT@N [9] uses TF and miRNA target prediction but does not use mRNA and miRNA expression data. Moreover, transcriptional regulation of miRNAs is often not included due to the challenge in reliable prediction of miRNA promoters [12,13]. In addition, chromatin immunoprecipitation with sequencing (ChIP-Seq) data for TFs experimentally characterize TF regulatory targets and have been combined and co-analyzed with mRNA expression profiling data [14], but usually only a small number of TFs were included. Multiple ChIP-Seq datasets from independent experiments were seldom compiled and incorporated into computational network inference. These limitations highlight the need for additional tools to systematically integrate genomic profiling datasets to better understand gene regulatory networks that govern complex biological systems.

In this study we developed a computational pipeline, InteGRaNet, to infer the gene regulatory network involved in Schwann cell response to injury. This network includes TF-mRNA, TF-miRNA and miRNA-mRNA regulatory interactions. This pipeline utilizes previously developed and new computational tools to integrate mRNA and miRNA expression data, ChIP-Seq data, and in-silico TF and miRNA target predictions. Starting with a set of genes and/or miRNAs obtained from expression profiling analysis, our approach initially constructs a network by connecting TFs to genes or miRNAs using TF targets identified from ChIP-Seq experiments. This network is then expanded to include additional regulatory targets of TFs and miRNAs by using genome-wide target prediction. We apply our computational pipeline to infer the Schwann cell injury response network and study the regulatory interactions around Egr2, a known key regulator of myelination. Furthermore, we study cooperative TF/miRNA regulation involved in the Schwann cell injury response network. This work demonstrates a systematic approach to integrate multiple genomic datasets and to infer TF and miRNA regulatory networks, which may be used to better understand coordinated gene regulation by TFs and miRNAs in complex biological systems.

## Results

### Overview of TF and miRNA regulatory network inference

**Figure 1:**
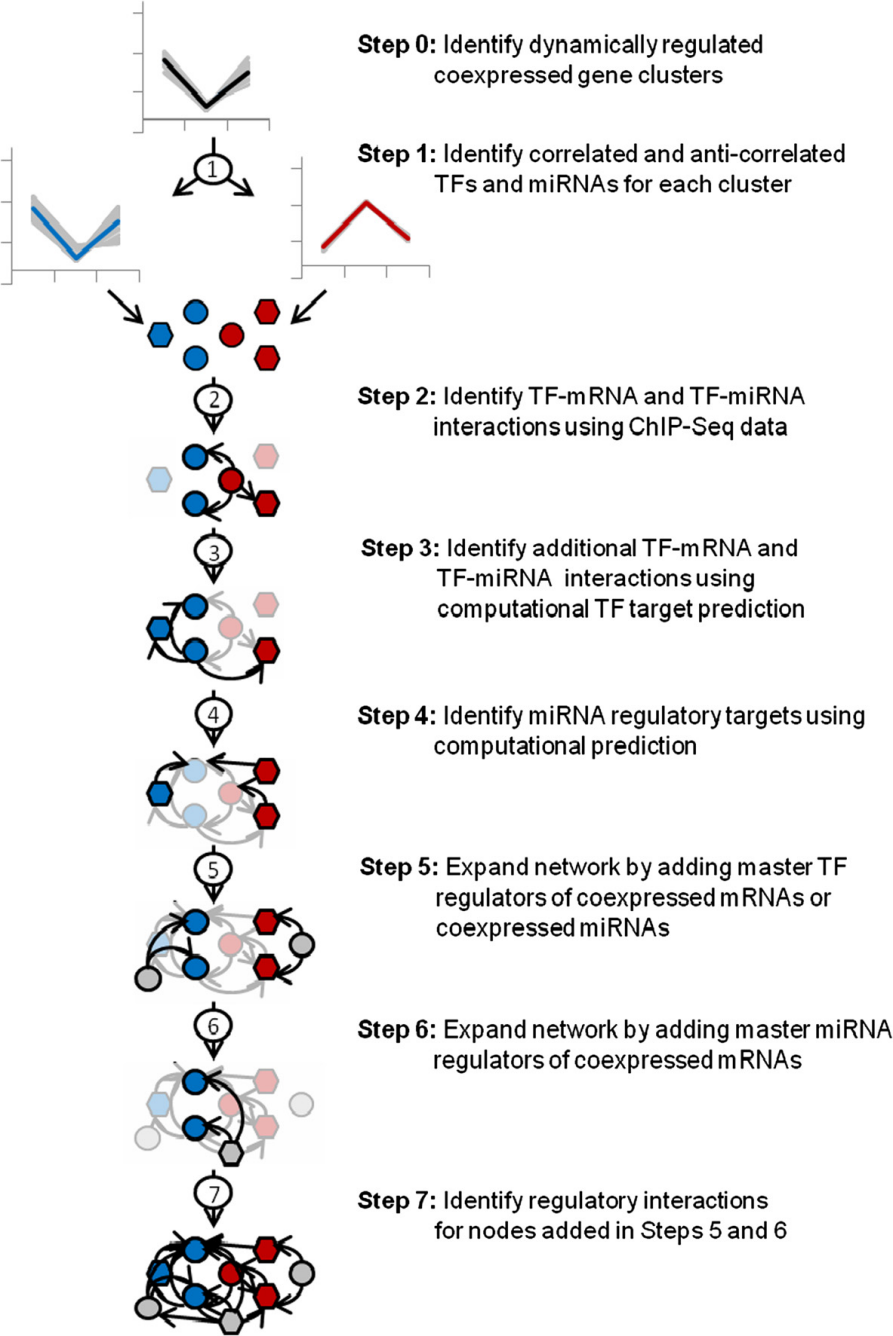
**Workflow of TF and miRNA regulatory network inference.** Starting with TFs and miRNAs that are correlated (blue profile) or anti-correlated (red profile) with a coexpressed gene cluster (black profile), sequential steps in this workflow illustrate how multiple types of experimental and computational information are integrated in regulatory network construction. In each intermediate step, nodes and interactions identified from earlier steps are placed in the background. Circles: TFs. Hexagons: miRNAs. Blue nodes: TFs or miRNAs that are correlated with the coexpressed gene cluster. Red nodes: TFs or miRNAs that are anti-correlated with the coexpressed gene cluster. Grey nodes: master TF or miRNA regulators of coexpressed genes.

We developed a systematic approach, termed InteGRaNet, for inferring TF and miRNA regulatory networks involved in SC injury response. This approach integrates mRNA and miRNA expression profiling data, chromatin immuneprecipitation with sequencing (ChIP-Seq) data, and computational TF and miRNA target predictions (Figure 1). Our approach first identified a set of genes involved in SC response to injury using previously collected mRNA and miRNA expression data. These genes consist of the initial set of genes included in the regulatory network. Briefly, co-expressed gene clusters that were dynamically regulated after SC injury, termed injury response gene clusters (IRGCs), were first identified (Figure 1, Step 0). TFs and miRNAs that were correlated or inversely correlated with the expression profiles of the IRGCs were identified as potential regulators of the IRGCs (Step 1). To identify TF-mRNA and TF-miRNA interactions, for TFs that have available ChIP-Seq data, regulatory interactions between TFs and their targets (mRNA or miRNA) were identified using ChIP-Seq peak locations (Step 2). For TFs that do not have ChIP-Seq data, we performed computational prediction to identify mRNAs or miRNAs that they regulate (Step 3). Next, miRNA-mRNA interactions were identified using computational miRNA target prediction (Step 4). All the identified interactions were organized into a regulatory network. This network was further expanded to include TFs that, although not correlated with IRGCs, were master regulators of coexpressed genes in each IRGC or that were master regulators of miRNAs coexpressed with each IRGC (Step 5). Similarly, miRNAs that were not correlated with IRGCs but were master regulators of genes in the IRGCs were identified (Step 6). These additional TFs and miRNAs were added to the network as nodes, and regulatory interactions between these regulators and other nodes already in the network were identified using interactions identified by ChIP-Seq data and computational predictions (Step 7). This final step completed the network inference. Each step in this approach to network construction is described in detail in the following sections.

### Identification of an initial set of genes involved in the SC injury response network

**Figure 2:**
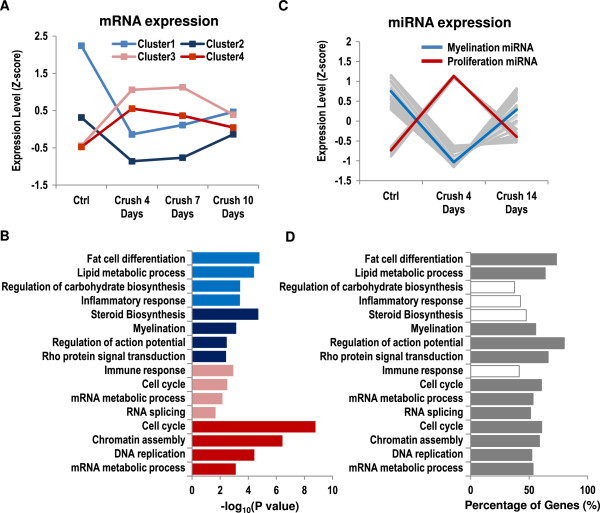
**Coexpressed gene clusters that are dynamically regulated during SC injury response.** These gene clusters are enriched for functional categories involved in SC myelination and proliferation, and many of the genes annotated in these categories are potential targets of dynamically regulated miRNAs. (**A**) Expression profiles of the four dynamically regulated injury response gene clusters (IRGCs). (**B**) Gene Ontology terms enriched in each of the four IRGCs. Color coding of bars matches that of the expression profiles shown in (**A**). (**C**) Expression profiles of miRNAs that are anti-correlated with clusters 1 or 2 (red) and miRNAs that are anti-correlated with clusters 3 or 4 (blue) and. (**D**) The percentage of genes in the enriched functional categories that have a seed sequence match to dynamically regulated miRNAs. Enriched categories that have at least 50 % of the genes that are potential targets of dynamically regulated miRNAs are highlighted with grey bars.

To identify a set of genes that are critical to SC injury response, we analyzed a published gene expression profiling dataset based on a mouse nerve injury model [15]. This study showed that core myelination genes were dynamically regulated and had distinct expression profiles during SC injury recovery and development [15]. Therefore, we performed unsupervised k-means clustering on the published mRNA expression data from the nerve injury experiment and from a SC development study [16]. Of all the genes profiled on the microarray, a total of 7,595 genes were expressed during at least one time point in the combined expression datasets, and we focused on gene clusters that were differentially expressed after nerve injury and that contained at least one known myelination genes (Additional file 1: Table S1) (see Methods). As a result, four coexpressed injury response gene clusters were identified (Figure 2A). Two of these clusters were downregulated immediately after crush and returned to pre-injury levels as the nerve regenertated (Clusters 1 and 2, referred to as myelination gene clusters, MGC). The other two clusters (Clusters 3 and 4, referred to as proliferation gene clusters, PGC) showed a reciprocal pattern of expression. These clusters contained 234, 160, 385, and 119 genes, respectively (Additional file 2: Table S2). The expression profiles of these clusters were similar to the previously identified profiles of myelination and proliferation genes using anchor genes [15].

To better understand the functions of these gene clusters, we performed Gene Ontology (GO) term enrichment analysis on the IRGCs. Consistent with initial dedifferentiation/proliferation and subsequent redifferentiation of SCs after nerve injury, we found that PGC genes (upregulated immediately after crush) were enriched for functional categories involved in cell proliferation, including cell cycle and chromatin assembly. MGC genes (donwregulated immediately after crush), on the other hand, were enriched for functional categories involved in SC differentiation, including lipid metabolic process and myelination (Figure 2B). The enriched categories in these clusters were therefore consistent with their expression pattern after nerve injury. Genes in the four IRGCs were used as the initial set of nodes for TF and miRNA network inference.

### Identification of potential TF and miRNA regulators in the SC injury response network

Transcriptional activators or repressors are likely to be correlated or anti-correlated with the expression of their target genes. Thus, to identify potential regulators of genes in the IRGCs we identified TFs that were correlated or inversely correlated with the expression profile of each IRGC. As a result, 6, 2, 23, and 2 TFs were found to have correlated expression with the four IRGCs, respectively. Three TFs, Cbfb, Taf9, and Mef2a, were found to have inversely correlated expression with Cluster 2. As shown in a recent study, miRNA regulators may be anti-correlated or correlated with the expression of their targets, functioning as either a reinforcer or a fine-tuner [17]. Thus, to identify miRNA regulators for genes in IRGCs, we analyzed the nerve miRNA expression measured before and after crush injury [6]. Comparing miRNA expression profiles with mRNA expression profiles, we found that, of the 87 miRNAs expressed in peripheral nerve, 17, 26, 6, and 6 miRNAs were correlated with the expression profile of the four IRGCs, respectively (Additional file 3: Table S3). 5 and 8 miRNAs were anti-correlated with the expression profile of Cluster 3 and Cluster 4 (Additional file 4: Table S4). Overall, this analysis identified 30 miRNAs that were expressed similar to MGC genes and 6 miRNAs that were expressed similar to PGC genes (Figure 2C, Additional file 5: Table S5). To check if the dynamically regulated miRNAs regulate key gene functions involved in myelination, we examined whether genes in the GO categories enriched in dynamically regulated IRGC genes (Figure 2B) were potential targets of the dynamically regulated miRNAs. For 12 of the 16 GO categories enriched in IRGCs, more than 50% of the annotated genes have a seed sequence match to at least one of these correlated or anti-correlated miRNAs (Figure 2D). This result suggests that these correlated or anti-correlated miRNAs are likely to be regulators of dynamically regulated genes in IRGCs.

### Identification of TF-mRNA and TF-miRNA interactions using ChIP-Seq data

To find TF-mRNA interactions among genes in the IRGCs, we first used experimentally validated TF targets characterized by ChIP-Seq analysis. Of all the 35 TFs identified in the IRGCs, we found 8 TFs whose genome-wide binding locations had been analyzed in 11 independently published ChIP-Seq datasets (Table 1). To identify more reliable TF-mRNA interactions we only used ChIP-Seq peaks that were located between -10 kb and +5 kb from the transcription start sites (TSS) of annotated genes.

**Table 1:** ChIP-Seq datasets used to identify TF regulatory interactions in the SC injury response network

**TF**	**Species**	**GEO accession number**	**Publication**	**Pubmed ID**	**Number of peaks**	**Number of targets**
Sox2	Mouse	GSE11431	Chen, 2008	18555785	4303	47
Klf4	Mouse	GSE11431	Chen, 2008	18555785	10296	387
E2f1	Mouse	GSE11431	Chen, 2008	18555785	17629	168
SOX2	Human	GSE23795	Fang, 2011	21211035	4883	41
Lmo2	Mouse	GSM552237	Hannah, 2011	21338655	9518	859
Cebpb	Mouse	GSM537985	Hannah, 2011	21338655	27683	5206
Fli1	Mouse	GSM552233	Hannah, 2011	21338655	19482	4217
Mef2a	Mouse	GSE21529	He, 2011	21415370	1337	75
NFKB1	Human	GSE19486	Kasowski, 2010	20299548	15522	2148
E2F4	Human	GSE21488	Lee, 2011	21247883	16246	1950
Cebpb	Mouse	GSE21314	Lefterova, 2010	20176806	24414	4435
STAT1	Human		Robertson, 2007	17558387	11004	2877
STAT1	Human		Schmid, 2010	20625510	4446	197
HIF1A	Human	GSE28352	Schodel, 2011	21447827	940	80

**Figure 3:**
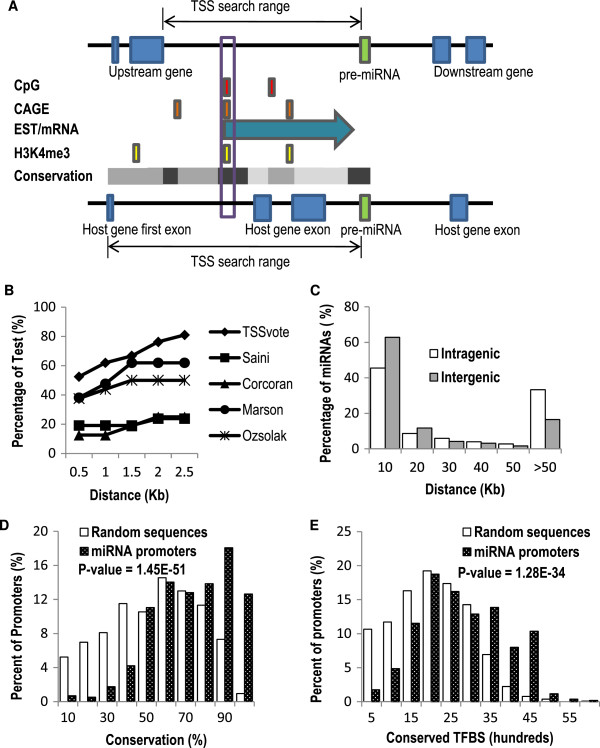
**A new computational method, TSSvote, predicts the transcription start site of miRNAs reliably.** (**A**) Illustration of the TSSvote algorithm. For intergenic miRNAs (top sequence bar), the TSS search range is defined as the genomic sequence between the end of the upstream gene and the start of the pre-miRNA. For intragenic miRNAs (bottom sequence bar), the TSS search range is between the TSS of the host gene and the start of the pre-miRNA. TSSvote calculates a score for each 100 bp sequence window within the TSS search range based on five supporting sequence features of TSS. The sequence window that is “voted” by the most features is predicted as the miRNA TSS. (**B**) Benchmarking computational methods for miRNA TSS prediction using a set of 21 experimentally validated miRNA TSS. For each method, the percentage of test cases where the distance between the predicted TSS and the true TSS is with a given distance is plotted. (**C**) The distribution of the distance between the predicted miRNA TSS and the annotated pre-miRNA. (**D**) The distribution of the percentage of sequence conservation in predicted miRNA promoters versus random sequences. (**E**) The distribution of the number of TF binding sites found in predicted miRNA promoters versus random sequences.

In addition to identification of TF-mRNA interactions, the genome-wide mapped reads of ChIP-Seq data may also be used to identify TF-miRNA interactions. Performance of such analysis depends on accurate characterization of pri-miRNA promoters. Several studies have developed computational methods to predict pri-miRNA TSSs using several promoter features [18-21]. However, given that many of these methods rely on limited experimental datasets, they tend to only predict TSSs for a subset of miRNAs. To address this limitation we developed a new voting algorithm (TSSvote) for predicting human and mouse miRNA TSSs, which uses a comprehensive set of transcription-related sequence features (Figure 3A, see Methods). These included mapping of known transcripts/ESTs, CpG islands, CAGE tags, H3K4me3 marks, and evolutionary conservation. Using TSSvote’s predicted pri-miRNA TSSs, we defined the promoter sequences of miRNAs as the genomic region between -5 kb and +1 kb from the predicted TSS. We used a shorter range compared to the analysis of mRNA because miRNAs genes are much shorter and 1 kb is usually enough to cover the entire gene and an additional downstream region. We then identified ChiP-Seq peaks of TFs located within these predicted miRNA promoters (for the TFs included in IRGCs). miRNAs that have TF ChIP-Seq peaks in their promoters are deemed as TF targets. Overall, using published ChiP-Seq datasets we identified 37 TF-mRNA and 15 TF-miRNA interactions for TFs in the IRGCs, connecting 23 TFs and 11 miRNAs. These interactions were added to the SC injury response network.

The overall performance of TSSvote is difficult to assess due to the limited number of experimentally validated miRNA transcription start sites. When the performance of TSSvote was tested by a compiled benchmark set of 21 experimentally determined human and mouse miRNA TSSs (Additional file 6: Table S6), TSSvote predicted 52% of these test TSSs within 500 bp and 81% of them within 2500 bp, outperforming all other currently available methods tested (as measured by the number of miRNA TSSs predicted within a given error range; Figure 3B). The predictions of TSSvote were further supported by the fact that a large proportion of miRNAs (63% of intergenic and 45% of intragenic miRNAs in mouse) were located within 10 kb from the pre-miRNA sequence (Figure 3C, Additional file 7: Table S7). Furthermore, the miRNA promoter sequences (as defined above) were more conserved than randomly selected intergenic sequences of the same length (Chi-square P-value=1.45E-51) (Figure 3D) and contained significantly more TF binding sites than random sequences (Chi-square P-value=1.28E-34) (Figure 3E).

### Additional TF target prediction using genome-wide TFBS enrichment analysis

Although ChIP-Seq data of TFs allowed the extraction of experimentally characterized TF-mRNA and TF-miRNA interactions, this information was only available for a subset of TFs. Moreover, because ChIP-Seq experiments might have been performed under different conditions, some transcriptional regulatory interactions critical to the SC injury response may not be identified. To address this shortcoming, we included computationally predicted transcriptional regulatory interactions based on an improved version of a previously developed statistical model for genome-wide TF binding site enrichment [22] (see Methods). Briefly, this approach calculated a binding probability score for each TF-gene pair using all the evolutionarily conserved TF binding sites (TFBS) in proximal promoters and evaluated a P-value using TFBS permutation (see Methods, Additional file 8: Figure S1). In this study, the model was improved by using a phylogenetic tree-based scoring function to incorporate evolutionary conservation information from more species. Using this model, we predicted 108,204 mouse and 132,516 human TF-mRNA interactions. By applying this model to the miRNA promoters predicted by TSSvote (Additional file 8: Figure S1) we also predicted a total of 2,658 mouse and 5,395 human TF-miRNA interactions. Using these predictions, we expanded the regulatory network to include 79 TF-TF interactions and 70 TF-miRNA interactions, connecting 34 TFs and 22 miRNAs.

**Figure 4:**
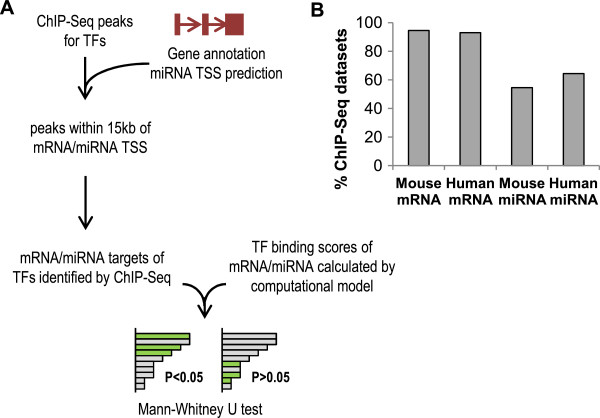
**Validating computational TF target prediction using the compendium of TF ChIP-Seq datasets.** (**A**) ChIP-Seq identified TF binding locations (peaks) within 15 kb around annotated mRNA transcription start sites (TSS) or predicted miRNA TSS were used to identify regulatory targets of TFs. Mann–Whitney U test was used to test if identified targets have higher binding scores calculated by the computational model. (**B**) Percent of ChIP-Seq datasets that were consistent with computational TF target prediction when tested using mouse or human mRNAs or miRNAs.

We validated this computational model for TF target prediction using TF regulatory targets identified by ChIP-Seq experiments. To perform this validation, we compiled a set of 120 independent published ChIP-Seq datasets for a total of 70 TFs (Additional file 9: Table S8), and we tested if the target genes identified by ChIP-Seq tended to have significantly higher scores for binding among all the genes in the genome, based on a Mann–Whitney U-test (Figure 4A). We found that ChIP-Seq identified mouse and human mRNA targets had significantly higher scores in 94% and 93% of the ChIP-Seq datasets, and ChIP-Seq identified mouse and human miRNA targets had significantly higher scores in 55% and 64% of the datasets (Figure 4B). This analysis showed that our computational TF-target prediction was consistent with experimental results, for both TF-mRNA and TF-miRNA regulation.

### Identification of miRNA-mRNA interactions using computational prediction

The previous ChIP-Seq data analysis and genome-wide TF target prediction identified TF-mRNA and TF-miRNA interactions. To identify miRNA-mRNA interactions we performed computational miRNA target prediction. A previous study showed that better performance of miRNA target prediction may be achieved by combining multiple currently available algorithms in order to reach reasonable specificity while minimizing loss of sensitivity [23]. Therefore we chose to combine TargetscanS [24] and pictar [25], which provides higher specificity, with miRanda [26], which provide higher sensitivity, to identify targets of miRNAs. Only targets of miRNAs predicted by at least two of these three methods were included in the network construction. Using this approach, we identified 57,980 mouse and 75,570 human miRNA-mRNA interactions. The average number of targets is 250 genes per miRNA, respectively, which is close to the speculated number of targets per miRNA [27]. Using this result, 43 miRNA-mRNA interactions were added to the SC injury response network.

### Expanding network to include master TF regulators of coexpressed mRNAs or miRNAs

Up to this step, the TFs included in the regulatory network were identified by their correlation or inverse correlation with the dynamically regulated IRGCs. However, master regulators of genes in the IRGCs may share a similar expression profile but with a lag time, or they may be constantly expressed throughout SC injury response while being modulated by mechanisms other than transcriptional control. These TFs will be missed by expression correlation-based discovery but could be identified as common regulators of genes in IRGCs based on enrichment of their TF binding sites. Therefore, we applied a previously developed tool, the Promoter Analysis Pipeline (PAP) [28], to identify curated TF binding sites that were enriched in the proximal promoter sequences of genes in each IRGC. As a result, we found several TFBS significantly enriched in genes in clusters 1, 2 and 4 based on a Bonferroni corrected P-value cutoff of 0.05 (Additional file 10: Table S9) (see Methods). These TFs included E2f1 and Nfyc that were correlated with the IRGCs and had been added to the network in Step 2. Remarkably, known functions of these TFs were consistent with the enriched GO terms for the corresponding gene clusters they regulate (e.g. Nfkb1 for inflammatory response, Egr2 for myelination, and E2f1 for cell cycle). Applying the same analysis to miRNAs correlated with the IRGCs, we found one TF, Spz1, whose binding sites were enriched in miRNAs correlated with cluster 2. These master TFs were added to the SC injury response network as additional nodes.

### Expanding network to include master miRNA regulators of coexpressed genes

Similar to TFs, common miRNA regulators of genes in the IRGCs may not have expression profiles that are tightly correlated or anti-correlated with their target genes. These miRNAs may be identified by the enrichment of their predicted target genes in the IRGCs. Thus, for each miRNA we calculated the hypergeometric P-value for its target enrichment (see Methods). As a result, we found 2, 2, and 3 miRNAs with a significant enrichment P-value for clusters 1, 2, and 3 respectively (Additional file 11: Table S10). Like the search of master TF regulators using TFBS enrichment, this analysis identified three miRNAs (let-7a, let-7f and miR-145) that were correlated with the expression of the IRGCs. Interestingly, miR-140, whose predicted targets were enriched in cluster 3, was not identified using expression correlation with IRGCs due to its low expression on microarray. However, qPCR experiments showed that miR-140 was indeed expressed in nerve and had an expression profile correlated with MGCs [6]. These results showed that analysis of miRNA target enrichment may identify miRNA regulators whose expression was not correlated or anti-correlated with its targets or miRNA regulators whose expression was not accurately measured on microarray.

### Expanding network to include regulatory interactions for additional regulators

**Figure 5:**
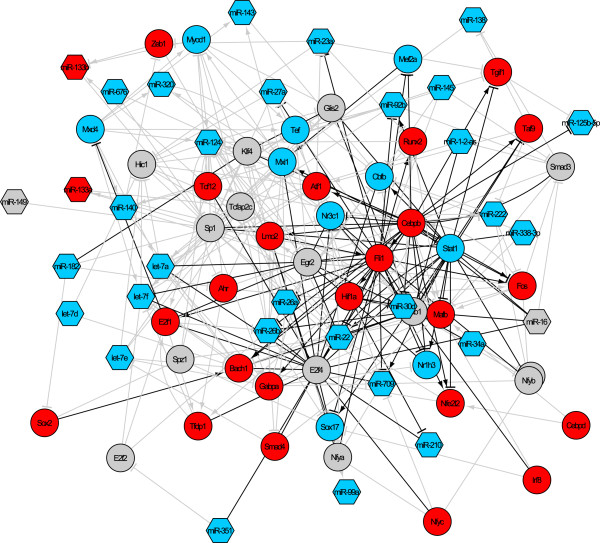
**The SC injury response regulatory network inferred by integrating experimental data and computational prediction.** Circles: TFs. Hexagons: miRNAs. Blue nodes: TFs or miRNAs correlated with myelination gene clusters. Red nodes: TFs or miRNAs correlated with proliferation gene clusters. Grey nodes: master TF or miRNA regulators of coexpressed genes. Black edges: regulatory interactions derived from TF ChIP-Seq data. Grey edges: regulatory interactions inferred by computational prediction. Arrowed edges: activation by TFs. T-shaped edges: repression by TFs or miRNAs.

After additional TF and miRNA regulators were identified as described above, they were first added to the network as additional nodes. Additional TF-mRNA, TF-miRNA, and miRNA-mRNA regulatory interactions between these regulators and other nodes in the network were then identified using ChIP-Seq data of TFs (Table 1) and computational TF and miRNA target predictions (Steps 2 and 3). The resulting TF and miRNA network involved in SC injury response included 146 TF-TF, 117 TF-miRNA, and 71 miRNA-mRNA interactions among TFs and miRNAs, connecting 48 TFs and 32 miRNAs (Figure 5).

### Availability of the InteGRaNet pipeline and datasets for network construction

The network construction pipeline we developed in this study including all the raw datasets is available to the public. These include 71,346 mouse and 64,367 human TF-mRNA interactions identified by the compendium of public ChIP-Seq data, high quality sets of 1,183 mouse and 1,511 human TF-miRNA interactions identified by miRNA TSS prediction and ChIP-Seq data, 108,204 mouse and 132,516 human computationally predicted TF-mRNA interactions, 2,658 mouse and 5,395 human computationally predicted TF-miRNA interactions, and 57,980 mouse and 75,570 human miRNA-mRNA interactions predicted by three algorithms. A Perl script can take a list of genes and miRNAs these data files as input and creates a network in a text format. These data files and the script are available upon request.

### Comparison to current algorithms for TF and miRNA network construction

To test the performance of our approach, we compared the InteGRaNet pipeline to currently available methods for constructing TF and miRNA regulatory networks, including GenMiR++ [8], MIR@NT@N [9], mirConnX [10], MAGIA [11] and EdgeExpressDB [12]. These methods use similar but different approaches and have different strengths and limitations. Of the six algorithms, MIR@NT@N, mirConnX, MAGIA and InteGRaNet predict all three types of interactions, i.e. TF-mRNA, TF-miRNA and miRNA-mRNA regulation. EdgeExpressDB only predicts TF-mRNA and miRNA-mRNA but not TF-miRNA interactions; GenMiR++ only infers miRNA-mRNA interactions using expression profiling data. Thus, while EdgeExpressDB and GenMiR++ can be used to predicted particular types of interactions, they are limited in comprehensive inference of comprehensive TF and miRNA networks. MIR@NT@N, mirConnX, MAGIA and InteGRaNet all use a pre-curated/pre-calculated set of TF and miRNA targets and combine this dataset with user inputted mRNA and miRNA expression data. Of these four methods, mirConnX allows users to change the weight of the predefined target dataset in network construction, whereas the other three do not provide this option. mirConnX and InteGRaNet use sophisticated statistical models to calculate TF and miRNA targets, whereas MIR@NT@N and MAGIA merely extract information from existing databases. Finally, EdgeExpressDB uses one human leukemia dataset to generate networks and does not allow users to use their own data to construct regulatory networks.

To benchmark these algorithms, we first compiled a set of known interactions using the GeneGO database (http://www.genego.com). GeneGO includes manually curated regulatory interactions from the literature. Using genes and miRNAs in our Schwann cell injury recovery network, the GeneGO database search returned a network that consisted of 871 connections, including 772 TF-mRNA, 30 TF-miRNA and 69 miRNA-mRNA interactions. Because these interactions were based on previous studies and were only a part of the complete SC injury network, interactions found by computational algorithms but not by GeneGO may not be false positives. Also, because GeneGO interactions were found in diverse biological systems and were not specific to Schwann cells, GeneGO interactions that were not found by computational algorithms might not be false negatives. For these reasons, it was difficult to evaluate the sensitivity and specificity of the algorithms.

**Figure 6:**
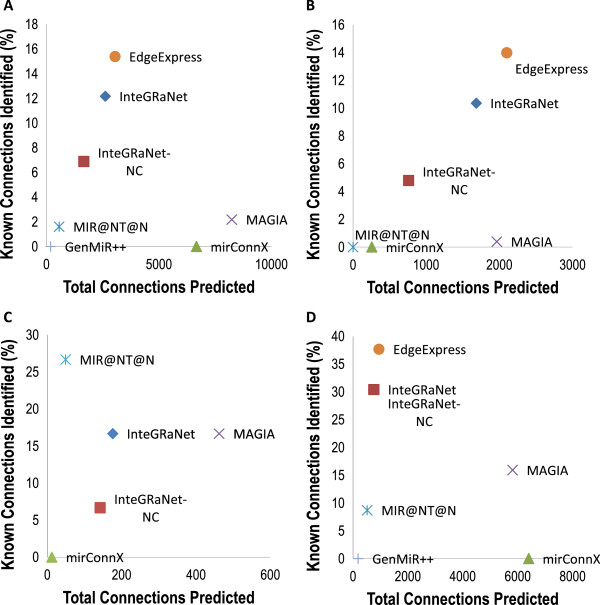
**Comparing the performance of InteGRaNet with current algorithms using known regulatory interactions in the literature.** For each algorithm, the total number of predicted interactions and the percentage of experimentally characterized interactions that were successfully predicted were plotted. InteGRaNet-NC: InteGRaNet without using ChIP-Seq data. (**A**) All interactions. (**B**) TF-mRNA interactions. (**C**) TF-miRNA interactions. (**D**) miRNA-mRNA interactions.

To reasonably test the performance of the algorithms, we adopted an approach previously used to benchmark the performance of miRNA target predicting programs [23]. We calculated the number of total predicted connections and the number of GeneGO validated connections that were predicted, and we compared these numbers between different algorithms. When interactions of all three types, TF-mRNA, TF-miRNA and miRNA-mRNA, were considered, EdgeExpressDB and InteGRaNet performed better than all other methods (Figure 6A). When each type of interactions was considered separately, EdgeExpressDB and InteGRaNet performed better than other methods in the TF-mRNA interactions and miRNA-mRNA connections (Figure 6B, 6D), and MIR@NT@N performed best in the TF-miRNA interactions (Figure 6C).

The GeneGO network also allowed us to evaluate the effect of including ChIP-Seq data in InteGRaNet. While the performance of InteGRaNet without ChIP-Seq data was similar to InteGRaNet with ChIP-Seq data in predicting TF-mRNA interactions (Figure 6B), ChIP-Seq data significantly improved the performance in predicting TF-miRNA interactions (Figure 6C). Predictions of miRNA-mRNA interactions did not use ChIP-Seq data and thus were not affected.

As a second approach to compare the performance of different algorithms, we calculated for each method the percentage of its predicted interactions that was also predicted by at least one other method (Table 2). Using this benchmark approach, InteGRaNet had the highest agreement rate in TF-mRNA and miRNA-mRNA interactions, and mirConnX had the highest agreement rate in TF-miRNA interactions. Overall, a total of 5858 TF-mRNA interactions were predicted by at least one of the six methods, 143 of which were predicted by at least two methods. A total of 691 TF-miRNA interactions were predicted by at least one method, 8 of which were predicted by two methods. A total of 13821 miRNA-mRNA interactions were predicted by at least one method, 582 of which were predicted by at least two methods and 182 of which were predicted by at least three methods. These interactions predicted by multiple algorithms would be good candidates for further study.

**Table 2:** Percentage of predicted interactions made by each algorithm that are also predicted by at least one other algorithm

	**InteGRaNet**	**mirConnX**	**EdgeExpress**	**GenMiR++**	**MAGIA**	**MIR@NT@N**
TF-mRNA	**1683 (8.4%)**	257 (0%)	2100 (6.6%)	NA	1963 (0.5%)	0 (0%)
TF-miRNA	176 (3.4%)	**12 (8.3%)**	NA	NA	462 (1.1%)	49 (8.2%)
miRNA-mRNA	**757 (56.8%)**	6397 (0.8%)	946 (47.4%)	188 (14.4%)	5810 (4.9%)	517 (25.1%)

The method that has the highest agreement rate in each type of interaction was highlighted in bold.

### Effect of model parameters on InteGRaNet performance

**Figure 7:**
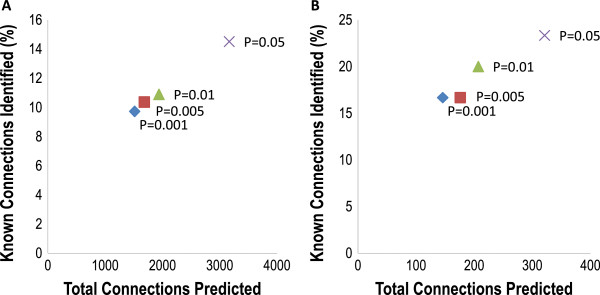
**Testing the performance of InteGRaNet using different statistical significance cutoffs.** For each cutoff selection, the total number of predicted interactions and the percentage of experimentally characterized interactions that were successfully predicted were plotted. (**A**) TF-mRNA interactions. (**B**) TF-miRNA interactions.

Using the GeneGO network, we compared the performance of InteGRaNet using different statistical significance cutoffs. The default P-value cutoff for predicting TF-mRNA and TF-miRNA interactions was 0.005. We compared the performance of InteGRaNet using four different P-value cutoffs, including 0.001, 0.005, 0.01 and 0.05. When predicting TF-mRNA interactions, all four cutoffs had similar performance in terms of the ratio between the total connections predicted and the number of predicted GeneGO interactions, with a cutoff of 0.005 performing slightly better than 0.001 and 0.01 (Figure 7A). When predicting TF-miRNA interactions, a less stringent cutoff of 0.005 predicted more interactions than a cutoff of 0.001 as expected but did not predict more GeneGO curated interactions (Figure 7B). Although this suggested that a P-value cutoff of 0.001 might be better, this benchmark result was based on a small set of 30 GeneGO TF-miRNA interactions.

### The Egr2 subnetwork revealed biological insights on regulation of myelination

**Figure 8:**
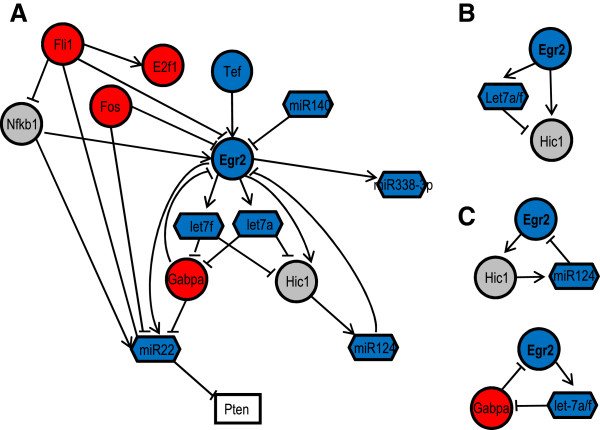
**The SC injury response regulatory subnetwork around Egr2 and Egr2 participating network motifs.** (**A**) Upstream regulators and downstream targets of Egr2 and regulatory interactions among them. (**B**) Two feedforward loops found in the Egr2 subnetwork. These feedforward loops are likely to maintain the expression level of Hic1 within a small functional range. (**C**) Three feedback loops found in the Egr2 subnetwork. These feedback loops may function as modulators of Egr2 expression levels. Circles: TFs. Hexagons: miRNAs. Blue nodes: TFs or miRNAs correlated with myelination gene clusters. Red nodes: TFs or miRNAs correlated with proliferation gene clusters. Grey nodes: master TF or miRNA regulators of coexpressed genes. White boxes: downstream target genes not included in the network. Arrowed edges: activation by TFs. T-shaped edges: repression by TFs or miRNAs.

To demonstrate the utility of the inferred TF and miRNA regulatory network, we studied regulatory interactions that involved a known key regulator of SC myelination, the early growth response 2 (Egr2/Krox-20) transcription factor. Egr2 is required for peripheral nerve myelin formation and maintenance and it is often mutated in patients with peripheral myelinopathies [29]. Specifically, we defined the Egr2-centered subnetwork as direct mRNA and miRNA targets of Egr2, TFs and miRNAs that directly regulate Egr2 and the interactions among them. In the Egr2 subnetwork, five TFs were predicted to regulate the expression of Egr2, two as activators (Nfkb1 and Tef) and three as repressors (Fli1, Gabpa and Fos) (Figure 8A). Interestingly, Nfkb1 and Fos, which forms the transcription factor complex AP-1 with c-Jun [30] have been previously associated with SC myelination [31] and dedifferentiation [32,33] respectively. In addition to TFs, two miRNAs, miR-140 and miR-124, were found to target Egr2. Additional experimental studies confirmed these interactions and showed that these two miRNAs contribute to the modulation of the expression of Egr2 (Additional file 12: Figure S2) [6]. Importantly, recent work from our laboratory showed that overexpression of miR-140 in SCs impaired their ability to form myelin *in vitro*, thus demonstrating a meaningful biological interaction between this miRNA and Egr2. Note that four of the five TFs regulating Egr2 expression were also found to modulate some of its targets, hence enhancing the ability of these TFs to regulate the myelination process.

Egr2 was in turn predicted to directly regulate the expression of the TF Hic1 and 3 miRNAs (let-7f, let-7a, and miR-22) (Figure 8A). These targets were particularly interesting as they may allow Egr2 to broadly regulate a number of genes and signaling cascades important for SC differentiation. For example, let-7a and let-7f, as well as other let-7 family members, are known to interact with a variety of targets to enhance cellular differentiation [34]. Similarly, the tumor suppressor miR-22 has been shown to target the 3’UTR of Pten and modulate Akt signaling, which critically determines the extent of SC myelination [35,36]. These results showed that our method to delineate the genetic networks driving the SC injury response elucidated Egr2 regulatory pathways that were consistent with current knowledge on the regulation of SC differentiation.

Examination of the Egr2 subnetwork also revealed that Egr2 participates in a number of potentially important regulatory network motifs [17,27,37]. In a network motif such as a coherent or incoherent feedforward loop, TFs and miRNAs cooperate to reinforce or modulate the transcriptional control of the common target gene. In the Egr2 subnetwork, Egr2 was found to participate in feedforward loops involving the tumor suppressor Hic1 and the miRNAs let-7a and let-7f (Figure 8B). The expression of Hic1 is not tightly correlated with Egr2, suggesting that the function of this feedforward loop is to maintain the expression level of Hic1 within a small range. In addition, we uncovered two feedback loops of Egr2 (Figure 8C). In the Egr2/Hic1/miR-124 negative feedback loop, Egr2 regulates Hic1, which induces miR-124 to inhibit the expression of Egr2. Using this feedback loop, Egr2 modulates its own expression with an oscillatory behavior. When Egr2 expression is too high or too low, it raises or lowers its own expression level through Hic1/miR-124. As a result, Egr2 expression is maintained within a range. Moreover, the expression of the mediator of this loop, Hic1, is also closely modulated by the Egr2/Let-7/Hic1 loop mentioned above, ensuring the robustness of this mechanism. Finally, Egr2 forms a positive feedback loop with let-7 and Gabpa. Egr2 activates let-7, which inhibits Gabpa, an inhibitor of Egr2. This loop allows Egr2 to assuage the inhibitory effect of Gabpa and increases its own expression. These feedforward and feedback loops cooperate to maintain the expression of Egr2 within a constant range. Together, these Egr2 network motifs suggest that the cooperation between miRNAs and TFs ensures rapid and robust transitions between the distinct differentiation states of SCs that are necessary to support nerve regeneration.

### TF and miRNA feedforward loops in the SC injury response network

**Figure 9:**
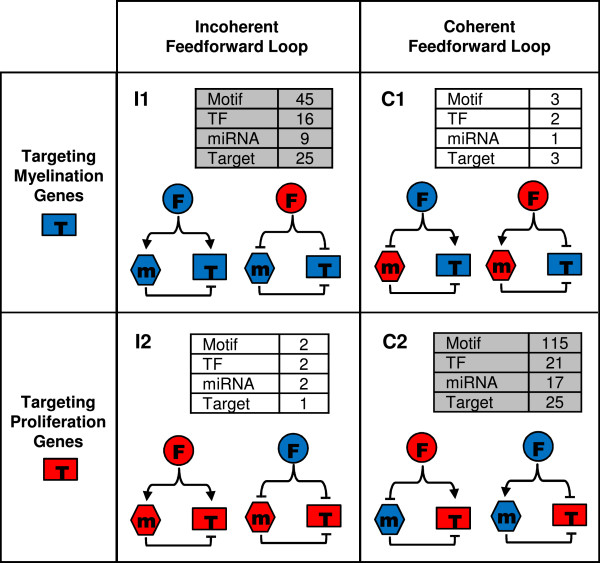
**Biased usage of incoherent and coherent feedforward loops in the SC injury response network.** Feedforward loops are divided into four categories according to the expression pattern of its target gene: I1: an incoherent loop regulating myelination genes, I2: an incoherent loop regulating proliferation genes, C1: a coherent loop regulating myelination genes, and C2: a coherent loop regulating proliferation genes. For each category, the number of network motifs in the SC injury response network and the number of TFs, miRNAs, and targets that participate in those motifs are shown, with more prevalent network motifs highlighted in grey. Circles: TFs. Hexagons: miRNAs. Rectangles: target mRNAs. Blue nodes: genes or miRNAs correlated with myelination gene clusters. Red nodes: genes or miRNAs correlated with proliferation gene clusters. Arrowed edges: activation by TFs. T-shaped edges: repression by TFs or miRNAs.

Recent studies have shown that coherent and incoherent loops of TFs and miRNAs are prevalent in the human genome [17]. The discovery of feedforward loops in the Egr2 subnetwork thus raised two interesting questions: whether coherent and incoherent feedforward loops are prevalent in the SC response network, and whether there exists a bias on the usage of coherent and incoherent loops to regulate genes in the SC injury response. To answer these questions, we searched for all the feedforward loops in the SC injury response network, and we analyzed these motifs in the context of the target gene’s expression profile (Figure 9, Additional file 13: Table S11). Namely, an incoherent or coherent feedforward loop may target genes that are correlated with the expression of differentiation/myelination genes (termed myelination genes) or with the expression of proliferation genes (termed proliferation genes), with the participating miRNA acting as a reinforcer or modulator (Figure 9). When we categorized all the regulatory loops based on the target gene’s expression and the type of the feedforward loop, we found that there is a significantly higher frequency of myelination genes in the incoherent loops than the coherent loops (Figure 9, I1 as opposed to C1). In contrast, there is a significantly higher frequency of proliferation genes in the coherent loops than the incoherent loops (Figure 9, C2 as opposed to I2). These results suggest that in SC injury response, regulation of myelination gene expression tends to be modulated or carefully controlled by miRNAs in incoherent feedforward loops, whereas regulation of proliferation gene expression tends to be reinforced by miRNAs in coherent feedforward loops. This analysis demonstrated that the inferred gene regulatory networks may provide new insights on the cooperative gene regulation by TF and miRNA in complex biological systems.

## Discussion

In this study, we developed a computational pipeline for TF and miRNA regulatory network inference that integrates expression profiling data of mRNAs and miRNAs, TF regulatory targets derived from ChIP-Seq data, and computational TF and miRNA target prediction. Our method takes a step-wise, bottom-up approach that starts with dynamically regulated co-expressed gene clusters as the basic network node set and sequentially adds TFs and miRNAs and their regulatory interactions to the network. By applying our approach to comprehensive delineation of the gene regulatory network underlying the SC response to nerve injury, we showed that this method allows inference of integrated gene regulation by TFs and miRNAs in complex biological settings. Our method was able to provide new insights into fundamental aspects of the SC regenerative response, indicating its potential to help elucidate the complexities of biological processes governed by intricate networks of TFs and miRNAs.

An important step in our approach was to use available Chip-Seq data to derive mRNA and miRNA targets of TFs. While a significant number of transcription factor ChIP-Seq data has been accumulated, only few studies have combined these datasets and used this resource to study transcriptional regulation of mRNAs [38], and no studies have co-analyzed these datasets to infer transcriptional regulation of miRNAs. This is partly due to the lack of reliable prediction of miRNA promoters. Our TSSvote algorithm incorporates several sequence features that imply transcription start sites and our method does not rely on experimental data that probe promoter usage, which may be dependent on the experimental conditions. Testing our algorithm using experimentally validated miRNA TSS sites showed that the accuracy of our prediction was within 2500 bp in 81% of the cases and the performance was better than current methods (Figure 3B). These predicted miRNA TSS allowed for the identification of ChIP-Seq peaks that were located within miRNA promoters. The identified miRNA promoters also allowed for the computational prediction of TFs that regulate miRNAs (Additional file 8: Figure S1). Our computational predictions are expected to be more accurate than previously reported methods [12,13] due to the more accurate miRNA promoter annotation and a more robust TF binding site analysis model.

A notable strength of our methods is that it integrates multiple types of experimental and computational data via a modular approach. Thus, individual components of the network inference pipeline may be improved or replaced separately, and additional information about TF or miRNA regulation may be added to the prediction model. For example, the computational prediction of TF targets may be further improved by incorporating epigenetic information [39]. Also, additional regulatory mechanisms, such as regulation by non-coding RNAs and by RNA binding proteins [40], may be added into the network once experimental data or computational prediction become available for these interactions.

The three major components in our pipeline include identification of TF targets using ChIP-Seq data, identification of TF targets using computational prediction and identification of miRNA target using computational prediction. All these components use a set of parameters and cutoffs to perform target identification or prediction, and their performance depends on the selection of cutoffs, with a lower cutoff generating more targets and a high cutoff generating fewer targets. While an arbitrary choice of cutoff is inevitable, we attempted to optimize our cutoff selection using independent datasets. For TF targets identified by ChIP-Seq data, the number of identified targets depended on the ChIP-Seq peak calling algorithm and its parameters. This performance of peak calling can be optimized but is beyond the scope of our manuscript. For computational prediction of TF targets, the P-value cutoff was selected and optimized in a previous publication by comparing to an independent study [22]. For computational prediction of miRNA targets, the cutoff was selected based on a previous estimate of the number of targets per miRNA [27].

A key component of our method was the prediction of TF-target interactions by computational models of TFBS enrichment. Regulatory networks inferred by large-scale genome-wide prediction methods like ours are often difficult to validate thoroughly and experimentally. However, our prediction method was based on a published statistical model that was validated using multiple datasets, including compiled sets of co-regulated genes and multiple ChIP-chip datasets [28]. Furthermore, the improved version of this model was compared to a large set of independent ChIP-Seq experiment data for 70 TFs and demonstrated good consistency for both TF-mRNA and TF-miRNA regulation. In addition, regulatory pathways identified in the subnetwork around Egr2, a well known transcription regulator of myelination, are consistent with current knowledge of regulation of SC myelination (Figure 8A). Remarkably, the post-transcriptional regulation of Egr2 by two miRNAs, miR-124 and miR-140, identified in our network were validated experimentally using luciferase assays (Additional file 12: Figure S2) [6]. These results suggest that our method produced an informative and reliable regulatory network for SC injury response.

To demonstrate that our network construction method may be used to gain insight on gene regulation and regulatory pathways in complex biological systems, we applied our method to study the TF and miRNA regulatory networks governing the SC injury response. This response involves the cycling of SCs between distinct differentiation states that support nerve regeneration. Proper cycling is accomplished through the reciprocal regulation of genes driving SC dedifferentiation and myelination respectively, through transcriptional control by TFs as well as post-transcriptional modulation by miRNAs [3-6]. The importance of transcriptional and post-transcriptional control in the SC injury response as well as the reciprocal nature of the genetic programs driving this process make SC injury recovery an ideal system for studying the cooperation of TF and miRNA mediated gene regulation. When we examined the SC injury response subnetwork around Egr2, a known key regulator of myelination, we found other regulators previously associated with SC differentiation. Furthermore, we found that Egr2 interacts with miRNAs in feedforward and feedback loops, which may be important for modulating the expression of both Egr2 and its targets.

miRNAs and TFs tend to cooperate in coherent or incoherent feed-forward loops, in which miRNAs may function as either a reinforcer or a modulator, to control the expression of a target gene [17,27,37]. In our analysis of network motifs in the SC injury response network, we found that genes involved in proliferation tend to be regulated by coherent loops, where their repression during SC injury response is reinforced by miRNAs. Genes involved in myelination, on the other hand, tend to be regulated by incoherent loops, where their activation during SC injury response is “fine-tuned” by miRNAs (Figure 9). This suggests that fast and precise timing of the activation/inactivation of genes associated with the immature state of SCs is most critical for the dedifferentiation of these glia after nerve injury. In contrast, proper remyelination seems not to require as carefully controlled timing of gene expression, but instead depend mostly on achieving precise functional levels of myelin-related proteins. This is particularly interesting because myelin formation and maintenance is very sensitive to gene dosage effects. In fact, both abnormally low or high levels of specific myelin proteins can cause peripheral neuropathy in humans [41].

## Conclusions

We present in this work a novel approach to TF and miRNA regulatory network inference. Our approach systematically integrates multiple types of experimental data and computational prediction on gene regulation and thus produces more reliable gene regulatory networks. Applying our approach to the SC injury response dataset demonstrates that our method may be used to gain new insight on gene regulation by TFs and miRNAs.

## Methods

### Identification of dynamically regulated SC injury response gene clusters (IRGCs)

SC mRNA expression profiling before and after crush and transection injury were performed using Affymetrix MU74Av2 chips in a previous study (Nagarajan et al., 2002). Gene expression levels were measured for uninjured nerves, on days 4, 7 and 10 after crush injury, and on days 1, 4, 7, and 10 after transection injury. Mouse gene expression profiling data during SC development were collected from an independent study [16]. This dataset profiled mRNA expression on days 0, 2, 4, and 10 after birth. Expression data were processed and normalized using Affymetrix MAS5 algorithm. A nerve-expressed gene was defined as one that was called present in at least one data point during SC injury response or development. k-means clustering was used to cluster genes based on the combined expression profiles of injury response and development. Gene clusters that contained known myelin genes and that were differentially expressed before and after crush injury based on a t-test were identified. Clusters with similar expression profiles based on the Pearson correlation coefficient were identified and merged. The average expression profile, i.e. the centroid, was calculated for each cluster. Nerve-expressed genes that were correlated with the centroids based on a Pearson correlation coefficient cutoff of 0.8 were identified as the final coexpressed IRGCs.

### Identification of miRNA regulators of SC injury response gene clusters

SC miRNA expression profiling before and after crush injury were performed using HTG Molecular qNPA miRNA microarrays in a previous study [6]. Expression of 1046 miRNAs was profiled using this microarray platform on days 0, 4 and 14 after crush injury. miRNA expression data were filtered using the following criteria: miRNAs that had an expression level lower than the average expression of the control miRNA probesets were removed from further analysis. miRNAs for which the expression of one duplicate probeset at all time points were significantly higher than that of the other duplicate probeset based on a Mann–Whitney U test were also removed from further analysis. After this filtering procedure, the average expression of the two duplicate probesets at each time point was used as the expression at that time point. miRNAs that were correlated or anti-correlated with the expression of IRGCs were identified using miRNA expression data and crush injury mRNA expression data on days 0, 4, and 10.

### Analysis of ChIP-Seq datasets

Publicly available ChIP-Seq datasets for human and mouse transcription factors were compiled and collected from literature search. Peak locations identified in the original studies were used if they are available. When peak locations were not available, Partek Genomic Suite with default parameters was used to identify peaks using raw alignment data. All peak locations were converted to genomic coordinates of human genome build hg18 or mouse genome build mm9. Peak locations of human datasets were then mapped to the mouse genome using UCSC’s liftover tool. Peaks that were located within the promoters of mRNAs or miRNAs were identified using NCBI’s gene annotation for mRNAs and computationally predicted miRNA TSS (see below). When peaks were mapped across species, only peaks that were located within proximal promoters and mapped to proximal promoters of orthologous genes (based on HomoloGene) were retained in the further analysis.

### Computational prediction of miRNA transcription start sites

To predict miRNA TSS, all human and mouse miRNAs were categorized as intergenic or intragenic miRNAs. Intragenic miRNAs were defined as miRNAs located between the start and end of a protein coding gene that is on the same strand (termed the host gene). miRNAs that are not intragenic were defined as intergenic. For intergenic miRNAs, the TSS search range was defined as the genomic sequence between the end of the upstream gene and the start of the pre-miRNA. For intragenic miRNAs, the TSS search range was defined as the genomic sequence between the start of the host gene and the start of the pre-miRNA. To predict miRNA TSS, a new algorithm, TSSvote, was developed to score each 100 bp window within the TSS search range based on transcription related sequence features. Mapping locations of known transcripts or ESTs and CpG islands were downloaded from the UCSC genome browser. CAGE tags were downloaded from the FANTOM project [42]. H3K4me3 histon modification marks were collected from a compiled set of H3K4me3 ChIP-Seq studies [43-51]. Conservation score was calculated as the number of aligned species in the 100 bp sequence window divided by the number of species in which the pre-miRNA was conserved. Using these sequence features, TSSvote calculated the score of each sequence window by *score* = 2*δ*_*transcript*/*EST*_ + *δ*_*CpG*_ + *δ*_*CAGE*_ + *δ*_*H*3*K*4*me*3_ + *conservation* where *δ*_*feature*_ equals one if a given feature is located within the sequence window. Otherwise, *δ*_*feature*_ equals zero. For each miRNA, the sequence window within the TSS search range that had the highest score was predicted as the miRNA TSS. When multiple sequence windows had the same score, the sequence window closest to the miRNA was assigned as the predicted TSS.

### Computational prediction of TF regulatory targets

To predict TF regulatory targets, we applied a previously developed computational model of transcription factor binding site (TFBS) enrichment [22] with several extended features, including more TF binding models and an improved phylogenetic model for TFBS conservation. Briefly, multiple sequence alignments of ten vertebrates, whose genomes were completely sequenced with a good coverage (>6x), were obtained from the UCSC genome browser download site. Using NCBI’s mouse genome annotation (build 37.1), for each mouse gene the multiple alignments of genomic sequence from -100 kb of the TSS to the end of the gene itself were extracted. Within this range, the sequence between -10 kb and +5 kb of the TSS and the sequence regions that have a regulatory potential (RP) score [52] larger than 0.1 were identified and collected as the TFBS search space. To search for TFBS, a total of 867 vertebrate position weight matrix models (PWMs) of TFs were compiled from the TRANSFAC [53], JASPAR [54], and UniProbe [55] databases. Using these PWMs, putative TFBS were identified in the TFBS search space using the program patser with the default score cutoff, and the evolutionary conservation of each site was determined using multiple sequence alignments.

The original model [22] only considered TFBS conserved in human, mouse and rat. Therefore, transcriptional regulation based on non-conserved sites was not accurately modeled, and the regulation of non-conserved genes was neglected. To overcome these limitations of the original model, we developed a phylogenetic tree-based scoring function to weight the contribution of each TFBS to the overall score by their evolutionary conservation. Namely, for each TF-gene pair, the phylogenetically weighted probability score of binding was calculated as

$$\sum_{x\in X}w_{x}\text{exp}\left(s_{x}\right)$$

where X is the collection of all sites, s_x_ is the PWM score of binding site x and w_x_ is the total phylogenetic tree branch length of all the species in which binding site x is conserved, based on a previously published tree [56]. Note that in this scoring formula the common branch length shared by two close species was only counted once. In this model, a site that is conserved in a distantly related species will gain a higher weight than one conserved in a closely related species.

Using this scoring model, the probability score for binding and the P-value were calculated based on all the identified TFBSs. Because the consolidated database of TF binding weight matrix models may have multiple models for the same TF, the bias in P-value calculation implanted by multiple hypothesis testing was removed by performing a Bonferroni correction on the raw P-value for each individual weight matrix of the same TF. Regulatory targets of TFs were identified using an adjusted P-value cutoff of 0.005, which was determined by comparing the number of computationally predicted targets to the number of ChIP-Seq identified targets for available TFs. The same analysis was applied to identify TFs that regulate miRNAs using miRNA promoters, which were defined as the sequence between -5 kb and +1 kb of the miRNA TSS predicted by TSSvote. For more details on the computational model please refer to the original paper that described the model [22].

### Computational prediction of miRNA regulatory targets

We combined miRNA target predicted by three algorithms, including TargetscanS [24], pictar [25], and miRanda [26]. miRNA targets predicted by TargetscanS were downloaded from http://www.targetscan.org/ (Release 4.2). miRNA targets predicted by pictar were downloaded from http://pictar.mdc-berlin.de/ and targets predicted by miRanda were downloaded from http://www.microrna.org (September 2008 release).

### Identification of master TF regulators of coexpressed mRNAs or miRNAs

Common TF regulators of coexpressed mRNAs were identified by the previously developed Promoter Analysis Pipeline (PAP) tool [28], which is available via a web interface or API at http://bioinformatics.wustl.edu/webTools/PromoterAnalysis.do. PAP searches for the enriched TF binding sites in the promoter sequences of coexpressed mRNAs or miRNAs. Briefly, an R-score was calculated for each gene in the mouse genome based on the ranking of the probability score for binding. Genes that are more likely to be regulated by a TF will have a higher R-score for that TF. For a set of coexpressed genes, the average of the R-scores of the member genes were calculated for each TF. The P-value for a given R-score was then calculated by using randomly selected gene clusters of the same size. A Bonferroni corrected P-value cutoff of 0.05 was used to identify TFs that had significantly higher average R-scores as common regulators. The same analysis was applied to identify common TF regulators of coexpressed miRNAs based on miRNA R-scores, which were calculated using the probability score for binding for miRNAs.

### Identification of master miRNA regulators of coexpressed mRNA genes

Common miRNA regulators of coexpressed mRNA genes were identified by the enrichment of miRNA targets in the coexpresed genes. The hypergeometric P-value for enrichment was calculated for a miRNA using the total number of nerve expressed genes that were predicted as targets of any miRNA (population size), the number of coexpressed mRNA genes (sample size), the number of nerve expressed genes that were predicted as targets of the miRNA (number of successes in population), and the number of coexpressed genes that were predicted as targets of the miRNA (number of successes in sample). Common miRNA regulators were identified using a hypergeometric P-value cutoff of 0.05.

### Experimental validation

Plasmids: pre-mir-124 was obtained through PCR amplification from genomic DNA. The resulting fragment was cloned between the BamHI and Nhe I sites in the miRNASelect pEP-MIR Cloning and Expression Vector (Cell Biolabs) using the InFusion HD cloning system (Clonetech) according to the manufacurer’s recommendations. Pre-mir-124 included the miRNA stem loop and ~100 nt of flanking sequence on either side. For luciferase assays, the 3’UTR region of *Egr2* was PCR amplified from genomic DNA using the following primers: *Egr2* 3’UTR: F, AAAGCT GCGCACTAGTGATGAAGCTCTGGCTGACACACCA; R, ATCCTTTATTAAGCTTACCA TAGTCAATAAGCCATCCAT. DNA fragments were cloned downstream of the luciferase gene between the HindIII and SpeI sites in the pMIR-REPORT miRNA Expression Reporter Vector (Ambion). The 3’UTR of *Egr2* lacking the miR-124 pad was cloned in an analogous manner. pRL-CMV Renilla Luciferase Reporter vector (promega) was used as a transfection control.

Luciferase assays: HEK293T cells were seeded at a density of 50,000 cells/well in 24 well plates in DMEM media (Invitrogen) supplemented with 10% fetal bovine serum (FBS), 2 mM L-glutamine. Cell were transfected 24 h later, with either a pEP-MIR vector expressing a pre-miRNA or with the pEP-mir Null control and with the pMIR-REPORT luciferase reporter vector containing the appropriate 3’UTR linked to luciferase. pRL-CMV Renilla Luciferase Reporter vector (Promega) was used as a transfection control. A total of 200 ng of plasmid DNA/well were transfected at a ratio of 50:1:0.5 (miRNA : luciferase reporter : transfection Ctrl). Cells were harvested 48 h post-transfection and assayed using a Dual-Luciferase Reporter Assay System (Promega) according to the manufacturer’s protocol.

## Abbreviations

SC: Schwann cells; IRGC: Injury response gene cluster; MGC: Myelination gene cluster; PGC: Proliferation gene cluster; GO: Gene Ontology; TSS: Transcription start site; TF: Transcription factor; TFBS: Transcription factor binding site; PAP: Promoter Analysis Pipeline; PWM: Position weight matrix

## Competing interests

The authors declare that they have no competing interests.

## Authors’ contributions

LC and AV designed the analysis, performed the analysis and wrote the manuscript. JEP performed a part of the ChIP-Seq data analysis. NV performed a part of the miRNA promoter analysis. JM and RN developed the project and critically read the manuscript. All authors read and approved the final manuscript.

## Supplementary Material

Additional file 1: Table S1 Known myelin genes and regulators. Click here for file

Additional file 2: Table S2 Genes in dynamically regulated SC injury response coexpressed gene clusters. Click here for file

Additional file 3: Table S3 miRNAs correlated with the expression of injury response gene clusters. Click here for file

Additional file 4: Table S4 miRNAs anti-correlated with the expression of SC injury response gene clusters. Click here for file

Additional file 5: Table S5 Dynamically regulated miRNAs cotegorized by correlation with differentiation/myelination or proliferation genes. Click here for file

Additional file 6: Table S6 List of experimentally characterized miRNA TSS used to test miRNA TSS prediction. Click here for file

Additional file 7: Table S7 TSS of human and mouse miRNAs predicted by TSSvote and supporting evidence. Click here for file

Additional file 8: Figure S1 Workflow of the computational method for predicting TFs that regulate mRNAs or miRNAs. The same computational model is used to predict TFs that regulate mRNAs using NCBI’s TSS annotation and to predict TFs that regulate miRNAs using computational TSS prediction. Click here for file

Additional file 9: Table S8 ChIP-Seq datasets used in validating computational TF target prediction. Click here for file

Additional file 10: Table S9 Enriched TF bind sites in genes in SC injury response gene clusters. Click here for file

Additional file 11: Table S10 Enriched miRNA binding sites in genes in SC injury response gene clusters. Click here for file

Additional file 12: Figure S2 Luciferase assays confirm a direct interaction between miR-124 and the 3’-UTR of Egr2. Overexpression of miR-124 but not of a Ctrl miRNA in HEK293T cells expressing a luciferase reporter construct carrying the 3’-UTR of Egr2 results in significantly decreased luciferase activity (p<0.05, two-tailed Student’s t-test). Mutating the predicted landing pad for miR-124 in the 3’-UTR of Egr2 disrupts the interaction between miR-124 and the Egr2 3’-UTR luciferase construct and restores luciferase activity. Click here for file

Additional file 13: Table S11 TF and miRNA regulatory network motifs in the SC injury response network. Click here for file
