# Supplementary figures and images for: An integrated approach to characterize transcription factor and microRNA regulatory networks involved in Schwann cell response to peripheral nerve injury
Source: BMC Genomics. 2013 Feb 6;14:84. doi: 10.1186/1471-2164-14-84 (PMC3599357; doi:10.1186/1471-2164-14-84)

# Supplementary Figure 1

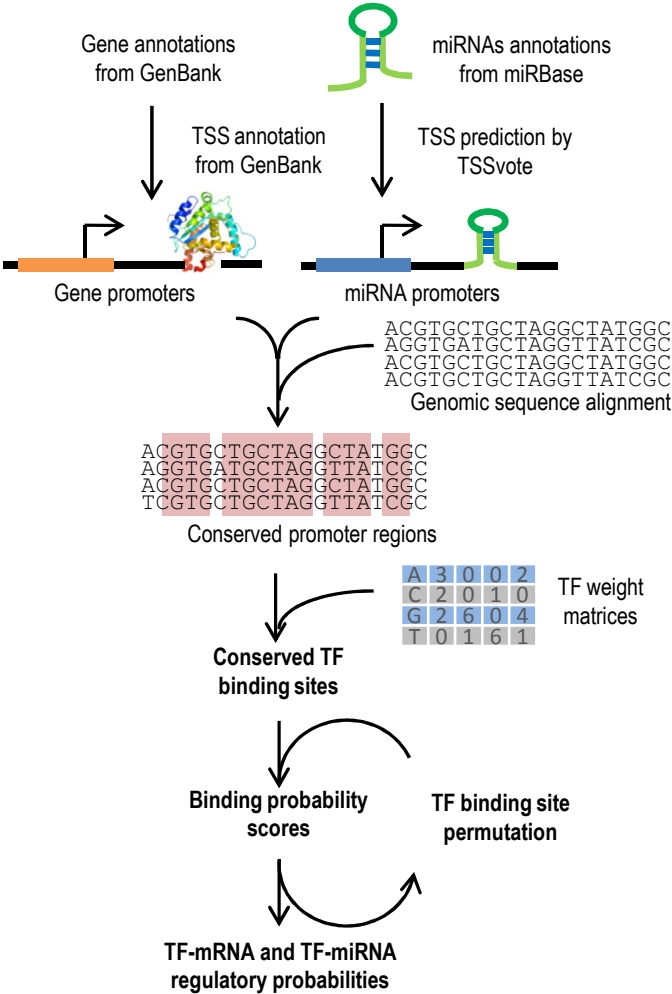

Supplement: Additional file 8: Figure S1 — Workflow of the computational method for predicting TFs that regulate mRNAs or miRNAs. The same computational model is used to predict TFs that regulate mRNAs using NCBI’s TSS annotation and to predict TFs that regulate miRNAs using computational TSS prediction. [file 1471-2164-14-84-S8.pdf]

Supplementary Figure 2

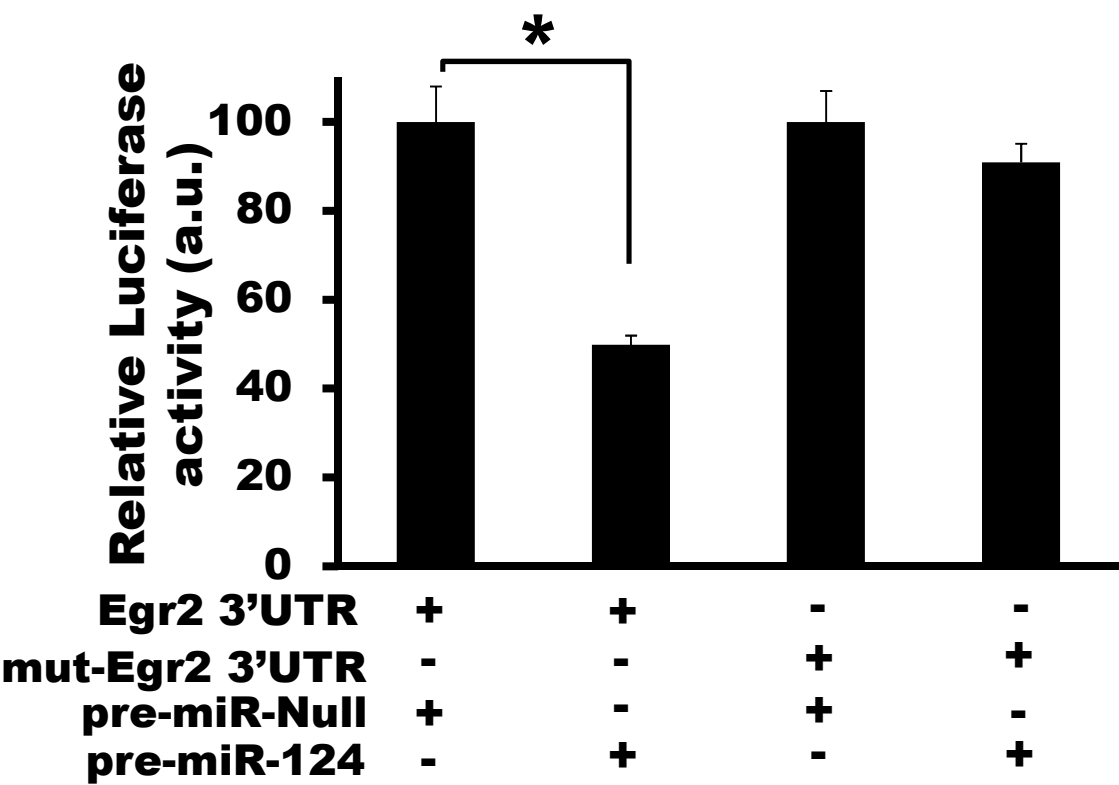

Supplement: Additional file 12: Figure S2 — Luciferase assays confirm a direct interaction between miR-124 and the 3’-UTR of Egr2. Overexpression of miR-124 but not of a Ctrl miRNA in HEK293T cells expressing a luciferase reporter construct carrying the 3’-UTR of Egr2 results in significantly decreased luciferase activity (p<0.05, two-tailed Student’s t-test). Mutating the predicted landing pad for miR-124 in the 3’-UTR of Egr2 disrupts the interaction between miR-124 and the Egr2 3’-UTR luciferase construct and restores luciferase activity. [file 1471-2164-14-84-S12.pdf]
